# An update on fractional picosecond laser treatment: histology and clinical applications

**DOI:** 10.1007/s10103-022-03704-y

**Published:** 2023-01-20

**Authors:** Yanjun Zhou, Michael R. Hamblin, Xiang Wen

**Affiliations:** 1 Department of Dermatology, West China Hospital, Sichuan University, No.37 Guoxue Road, Chengdu, 610041 China; 2 Laboratory of Dermatology, Clinical Institute of Inflammation and Immunology, Frontiers Science Center for Disease-Related Molecular Network, West China Hospital, Sichuan University, Chengdu, 610041 China; 3 Laser Research Centre, Faculty of Health Science, University of Johannesburg, Doornfontein, 2028 South Africa

**Keywords:** Picosecond laser, Fractional optical delivery systems, Histologic characteristics, Clinical applications

## Abstract

Picosecond lasers have a very short pulse duration and a high peak power density. When fractional optical delivery systems are attached to picosecond lasers, they generate an array of concentrated microspots with a high fluence surrounded by areas with a low fluence. This article discusses the histologic characteristics and clinical applications of fractional picosecond laser treatment. Fractional picosecond laser produces laser-induced optical breakdown (LIOB) and laser-induced cavitation (LIC) in the epidermis and dermis respectively, and can encourage skin regeneration and dermal remodeling. It has been shown that fractional picosecond laser has a positive effect on facial photoaging, enlarged facial pores, dyspigmentation, wrinkles, and atrophic scars. Further research is still needed to confirm the benefits of fractional picosecond lasers.

## Introduction

Laser treatment has long been used in dermatology. The wavelength governs the energy of the photons, while the pulse duration governs the time during which the laser delivers energy to the tissue, and the thermal relaxation time refers to the time required for the heated target tissue to reduce its absorbed energy by 50% through thermal diffusion. The target tissue undergoes specific thermal damage when the pulse duration is shorter than the thermal relaxation time [1]. Picosecond lasers can provide pulse durations between 300 and 900 picoseconds (10^–12^ s) [2]. In 2012, the FDA approved the first picosecond laser for skin applications (Picosure, Cynosure, Westford, Massachusetts) [1].

Regarding the wavelength, picosecond lasers include the picosecond alexandrite laser (755 nm) and the picosecond neodymium: yttrium‐aluminum‐garnet (Nd:YAG) laser (1064 nm), which delivers 730-nm and 785-nm laser light when used with a laser‐pumped handpiece, or 532 nm if frequency doubled [3].

For skin cosmetology, laser-tissue interactions include photothermolysis, photomechanical (photoacoustic) effect, photochemistry, and photobiomodulation (biostimulation). Photothermolysis involves tissue vaporization or melting through heating by lasers, including the carbon dioxide (CO_2_) laser or the erbium: yttrium–aluminum-garnet (Er:YAG) laser [1]. The photomechanical or photoacoustic effect occurs when an ultrafast temperature increase generated by a picosecond pulse causes a powerful acoustic shock wave after absorption by a tissue chromophore, producing a tensile stress beyond the tissue fracture threshold [2]. Photochemistry occurs when laser energy is absorbed by the tissue and triggers chemical reactions, which leads to the breaking of chemical bonds between the molecules, and then weakens and destroys the tissue [1]. Photobiomodulation refers to delivering energy to mitochondria, changing the permeability of cell membranes, stimulating fibroblasts to synthesize more collagen and elastin, or regulating the cell signal transduction pathway to upregulate or downregulate the expression of certain genes, rather than producing skin rejuvenation by the effects of tissue heating. Picosecond laser therapy differs from nanosecond laser therapy in that the pulse width is shorter and the peak power density is higher [4]. Nanosecond laser treatment relies mainly on photothermolysis rather than photomechanical effects; however, picosecond lasers rely mainly on photoacoustic destruction [5, 6]. This approach can enhance the energy transmitted to target cells within the lesion, and avoid thermal damage to surrounding tissues[6].

A variety of holographic or diffractive lens arrays can be used to produce fractional picosecond laser energy. These arrays allow the energy to be concentrated within laser microbeams, while neighboring tissue between the microspots is unharmed [2]. Commercially available fractional optical delivery technologies include, diffractive lens arrays (DLA), micro-lens arrays (MLA), and holographic optical arrays [7]. Different optical arrays with different spot sizes have been applied in various picosecond laser devices. DLA technology is employed in the 755-nm alexandrite picosecond laser, and involves closely packed individual hexagonal lenses with a 500 μm pitch [8]. The DLA produces a hexagonal pattern of high-intensity spots surrounded by low-intensity background [8]. The 532-nm and 1064-nm picosecond Nd:YAG laser employ a MLA with a spot size of 4 mm to produce high-intensity zones of tissue damage while preserving the remainder of the surrounding tissue unaffected [9]. This holographic optical array (PicoWay Resolve, Candela) delivers an array of 100 microbeams over a 6 mm × 6 mm area [10].

These systems all employ fractional optical delivery devices attached to picosecond lasers [11]. In this article, we review the histological characteristics and clinical applications of fractional picosecond laser treatment (Table 1).

**Table 1:** Single fractional picosecond laser treatment

Disease	Laser	Therapy parameters	Fitzpatrick scale	Number of cases	Clinical outcome	Adverse events	Reference
Facial rejuvenation	755-nm picosecond laser with DLA	8 mm spot, fluence of 0.4 J/cm^2^, 5 Hz pulse rate or 6 mm spot, fluence of 0.71 J/cm^2^, 10 Hz pulse for six treatments at 4-week intervals	III–IV	18	Skin texture and dyspigmentation scores improved significantly; however, in pore size or wrinkles, there was no significant improvements	Transient erythema and edema	[20]
	755-nm picosecond alexandrite lasers with DLA	8-mm spot, fluence of 0.4 J/cm^2^, 750 ps pulse duration, 10 Hz pulse repetition for 3–5 treatment sessions at 4-week intervals	IV	10	Pores had improved at 12- and 20-week follow-up, while at 1-year follow-up, the score of pigmentation showed 38% improvement, and wrinkles improved significantly.	Mild erythema, itchiness, desquamation	[21]
	755 nm picosecond alexandrite laser with a DLA	8‐mm spot, 0.4 J/cm^2^, 10 Hz pulse repetition, and 750 ps pulse duration for 10 sessions at 2-week intervals	III–IV III (*n* = 2); IV (*n* = 8)	11 (10 patients completed)	Pigmentation: compared with the control side, the improvement of the treated side reached statistical significance at follow‐up Wrinkles: the trend of improvement did not reach statistical significance at 6‐month follow‐ups	Transient erythema and edema, slight PIH	[22]
	755 nm picosecond laser with DLA	8 mm spot, 10 Hz, 0.4 J/cm^2^ or 10 mm spot, 10 Hz, 0.25 J/cm^2^ in just one treatment session	III–IV III (*n* = 29); IV (*n* = 17)	46	Wrinkles improved significantly, over half of patients showed improvement in pore size	Mild hyperpigmentation	[23]
	Combination of the 1064 and 532-nm picosecond laser with a fractional handpiece	Total pulse energy: 350 mJ for the 1064-nm wavelength, 250 mJ for the 532-nm wavelength The 532-nm component followed the 1064-nm portion	I–III I (*n* = 2); II (*n* = 12); III (*n* = 4)	18 (10 subjects completed)	At 1-month follow-up, 79% of patients had mild‐to‐moderate wrinkle improvement. 93%, 78%, and 87% of patients showed mild to significant mottled pigmentation improvement at 1-month, 3-month, and 6-month follow-up	Trace erythema	[24]
Wrinkles	755 nm alexandrite picosecond laser with DLA	6 mm spot, fluence of 0.57 J/cm2, 750 ps pulse duration, 10 Hz repetition rate for 5 treatments at 4-week intervals. Standard half face treatment: an average of 3301 + / − 155 pulses, the other half of face: an average of 5867 + / − 500 pulses	II–IV	4	Two treated sides had no significant difference. The standard pulse side had good to excellent improvement, and the high pulse side had very good to excellent improvement	Erythema and edema	[25]
	755 nm alexandrite picosecond laser with DLA	6 mm spot, fluence of 0.71 J/ cm^2^, 750 ps pulse duration, 10 Hz pulse repetition for 4 treatments at 1 month intervals	I–IV I (*n* = 4); II and III (*n* = 34); IV (*n* = 2)	40	At the 6-month follow-up, the average Fitzpatrick wrinkle score was 3.47 (the baseline was 5.48). 31.6% of individuals were very much improved, 28.9% were much improved, and 28.9% were improved at 6-month follow-up.	Transient erythema, edema, bruising	[26]
Dyspigmentation (melasma)	755 nm alexandrite picosecond laser with DLA	8-mm spot size, fluence of 0.4 J/cm^2^, 750 ps for three treatment sessions at 4- to 6-week intervals	IV	20	The mean MASI score improved to 6.9 ± 3.7 after 3 sessions treatment with the baseline was 9.4 ± 4.7	Erythema, pruritus, scaling, and only one developed mild PIH	[27]
	Fractional 1064-nm picosecond laser	450 ps pulses with a maximum microbeam energy per pulse of 3 mJ	III–IV III (*n* = 2) IV (*n* = 8)	10	Compared with baseline, 7 patients showed moderate to marked improvement at 6 weeks post-treatment, and 5 patients showed sustained improvement	Erythema, edema, hyper/hypopigmentation	[28]
Dyspigmentation (PIH)	755-nm alexandrite picosecond laser with DLA	8 mm spot, fluence of 0.4 J/cm^2^, 750 ps, 10 Hz pulse repetition for three treatments at 1- to 2‐month intervals	III	1	At the 3-year follow-up, the PIH lesions had 50–75% improvement	Deepening of local skin lesions	[4]
Facial pores	Picosecond 1064-nm laser with MLA	8 mm spot, 0.8 J/cm^2^, 10 Hz for one session	III–IV	59	Compared to baseline, the number of enlarge pores decreased by 15.13%, while the diameter of face pores did not alter much	Mild erythema and folliculitis	[29]
	1064‐nm picosecond laser with MLA	8 mm spot, fluence of 0.8 J/cm^2^, 5 Hz pulse repetition for three treatments at 4‐week intervals	III–IV III (*n* = 19) IV (*n* = 6)	25	Pore volumes had a significant reduction, with average pore size had shrunk by 30% at 6-month follow-up	Moderate erythema,mild‐to‐moderate swelling, acneiform eruptions	[30]
Atrophic acne scars	755 nm alexandrite picosecond laser with DLA	fluence of 0.71 J/cm^2^, 6 mm spot, 750 ps pulse width, repetition rate of 5 Hz for 6 sessions at 4 to 8 weeks intervals	I–V I (*n* = 1) II (*n* = 7); III (*n* = 6); IV (*n* = 3)	20 (17 patients completed)	Patients were satisfied with overall appearance and texture. scar volume improved 24.3% at the sixth treatment session	Transient erythema and edema	[15]
	755 nm alexandrite picosecond laser with DLA	6 mm spot, fluence of 0.57 J/cm^2^, 750 ps pulse duration, 10 Hz repetition rate for 5 treatments at 4-week intervals. standard half face treatment: an average of 3301 + / − 155 pulses, the other half of face: an average of 5867 + / − 500 pulses	II-IV	3	Two treated sides had no significant difference. The standard pulse side had good to excellent improvement, and the high pulse side had very good to excellent improvement	Mild transient PIH, erythema, and edema	[25]
	755-nm diffractive lens picosecond laser	Different sessions according to the patient’s condition	Not mentioned	3	Two cases improved > 75%, one improved 50–75% in skin texture	No	[31]
	755 nm alexandrite picosecond laser with DLA	6 mm spot, fluence of 0.71 J/cm^2^, 5‐Hz pulse repetition, and 750 ps pulse duration for 3 sessions at 4‐ to 6‐week intervals	III–IV III (*n* = 5); IV (*n* = 15)	20	After three picosecond laser treatments, the texture of acne scars and post-inflammatory erythema greatly improved	Transient and mild erythema, edema, and scabbing	[32]
	1064 nm picosecond laser with MLA	Fluence of 1.0 J/cm^2^, 8 mm spot, repetition rate of 10 Hz for 6 treatments at 1-month intervals	III–IV	26	Skin surface roughness and scar showed significant improvement	Mild‐to‐moderate erythema and swelling; transient PIH	[33]
	Picosecond 1064-nm laser with MLA	8 mm spot, 0.8 J/cm^2^, 10 Hz for one treatment	III–IV	59	Compared to baseline, acne scar volume reduced significantly	Mild erythema and folliculitis	[29]
Striae distensae	1064 nm picosecond laser and MLA	Fluence of 0.6 J/cm^2^, 8 mm spot, 750 ps pulse width, repetition rate of 10 Hz for 4 sessions at 4-week intervals	IV–V	20	Significant improvement in the skin texture; 6 patients improved 51–75%, and 12 patients improved 25–50% in striae with the 6-month follow-up	Transient PIH in two patients	[34]

## Histological characteristics and mechanisms

Intradermal laser-induced cavitation (LIC) and laser-induced optical breakdown (LIOB) both produce tiny lesions within the dermis and the epidermis [12]. The mechanism of tissue breakdown induced by picosecond laser relies on free electrons, generated by either thermionic emission or chromophore-independent multiphoton absorption [12, 13]. Subsequently in the focus region, the electron density increases to form a plasma, which can more effectively absorb the remaining energy of the laser pulses. The plasma then expands driving the shock wave, and finally the expansion of the vaporized material creates cavitation bubbles, which spread outward into the nearby tissue, resulting in a microcavitation structural response [8, 11].

LIOB and LIC both appear to encourage local skin regeneration and skin remodeling. Habbema et al. [14] observed new collagen production close to the sites of optical breakdown. Similarly, in a study by Brauer [15], increased density and elongation of elastin fibers in the dermis, as well as mucin and collagen III deposition, were observed during the 3-month follow-up in patients treated with 755-nm picosecond laser using a DLA. In response to the LIOB damage in the epidermis, the keratinocytes release a variety of cytokines, chemokines, and growth factors [16]. The rapid development of vacuoles associated with the creation of a LIOB can produce skin pressure fluctuations, which could alter cell signaling and result in dermal remodeling [8]. A study by Ahn et al. has also shown that extravasation of red blood cells could be observed in the papillary dermis after treatment with 1064-nm picosecond laser with MLA, and it was suggested that LIOB could disrupt the neighboring blood vessels to produce a dermal remodeling effect [17]. A similar phenomenon was observed after treatment with a 755-nm picosecond laser with DLA [8].

One study compared the skin thermal effects of laser treatment with fractional or flat optics, and the results showed that compared with flat optics, treatment with fractional optics resulted in more obvious localized epidermal necrosis, which produced significant thermal and clinical effects [18].

It has been found that the depth of a laser-induced lesion and the corresponding laser energy level show an inverse correlation [12]. Higher energy levels result in more superficial intraepidermal LIOBs, whereas lower energy settings result in deeper intradermal LICs [12]. The explanation for this phenomenon is that in intradermal LICs, the low-energy laser beam cannot trigger the threshold for the formation of LIOBs, instead when the high-energy laser beam is greater than the threshold, the energy is focused into LIOBs [12, 19].

The number and size of LIOBs in the epidermis appears to be correlated with the fluence and the number of passes, according to a study by Chang [7]. Similarly, another study showed that the size and number of LIOBs in the epidermis increased as the fluence or the amount of melanin in the skin increased. It was also shown that treatment with higher energy settings produced larger numbers and sizes of LIOBs [8].

## Skin rejuvenation

**Fig. 1:**
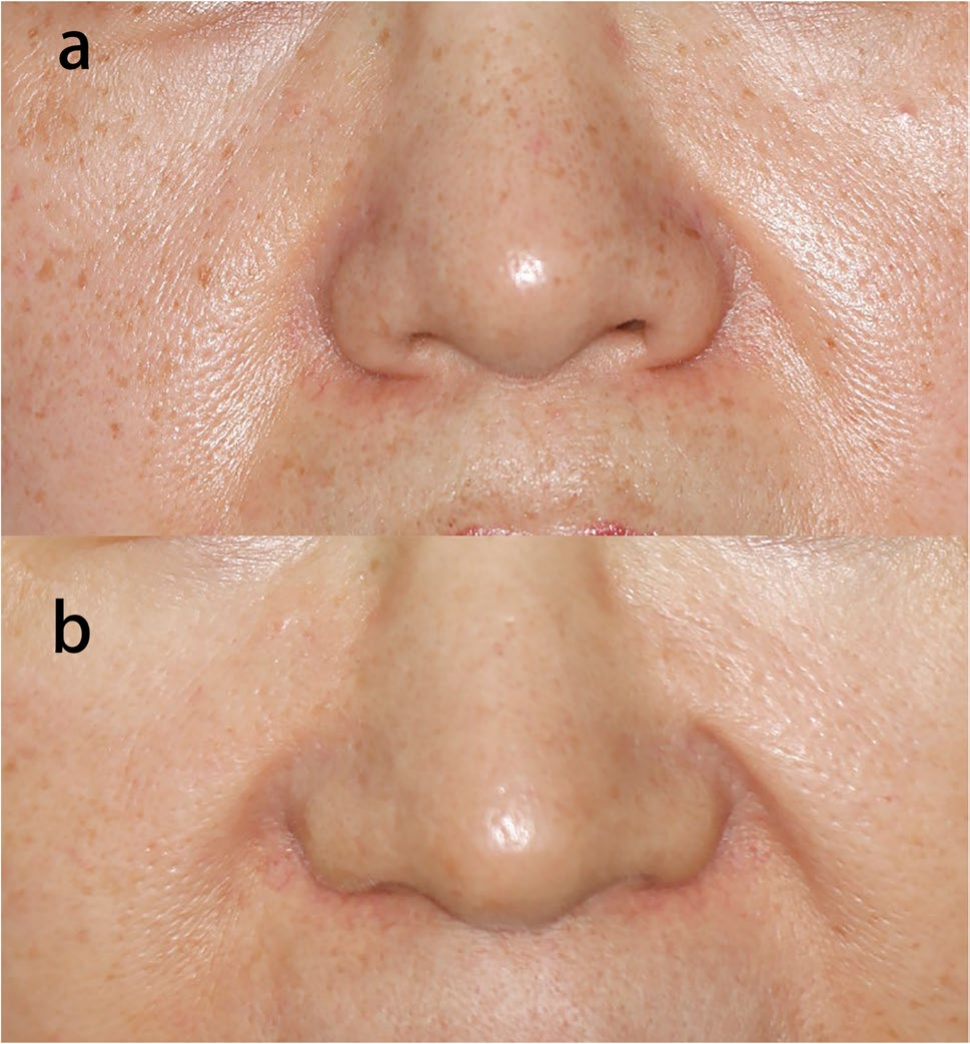
**A** Photo of a patient with facial photoaging; **b** 7 months after one fractional picosecond laser treatment (wavelength 755 nm, focus mode, energy 0.71 J/cm2, frequency 10 Hz, spot size 6 mm)

Long-term exposure to ultraviolet radiation can cause facial photoaging (Fig. 1). Irregularities in skin texture, skin laxity, dyspigmentation, wrinkles, and enlarged pores are all signs of skin photoaging [20]. Effective skin rejuvenation is becoming more popular as a clinical treatment, and some studies have used fractional picosecond laser for facial rejuvenation.

Wat et al. reported the first prospective trial to assess the safety and effectiveness of a 755-nm picosecond laser coupled DLA in Chinese patients with photoaging [20]. After six treatment sessions, the skin texture and dyspigmentation scores showed statistically significant improvement; however, there was no significant improvement in pore size or wrinkles [20]. Similarly, in a study by Lin, 755-nm picosecond laser with DLA was used in Asian patients, and the result showed pores and pigmentation had improved at 12 and 20 week follow-up, while at the 1-year follow-up, the improvement in pores had regressed, although pigmentation showed 38% improvement, and wrinkles also improved significantly [21].

Yu et al. treated patients with facial photoaging with 755 nm picosecond alexandrite laser coupled DLA using a fluence of 0.4 J/cm^2^, an 8-mm spot, and 10 Hz pulse rate for 10 sessions at 2-week intervals. At the 6-month follow-up, there was no significant improvement in wrinkles, while the improvement in pigmentation reached statistical significance compared with the control side [22]. In another study in 46 Asian patients, all patients showed statistically significant improvement in wrinkles, and the pore size improved in more than half the patients after one session of 755 nm picosecond laser with DLA [23]. Ross et al. found that fractional picosecond laser was well tolerated, and wrinkles and improvement in pigmentation were scored as mild to moderate after treatment with the 532- and 1064-nm picosecond laser equipped with a fractional handpiece [24].

The above studies have shown that the fractional laser systems have overall good effects on facial photoaging, while the benefits observed in wrinkles, dyspigmentation, and pore size are considered promising.

### Wrinkles

Dierickx et al. used a 755-nm alexandrite picosecond laser with DLA to treat seven cases of facial wrinkles in a split face study [25]. One half of the face received the standard protocol number of pulses while the other side received a higher number of pulses, and both sides showed comparable facial rejuvenation [25]. In another study by Weiss, a picosecond 755-nm alexandrite laser with DLA (fluence of 0.71 J/cm^2^, 6 mm spot size, 4 treatments at 1 month intervals) was used to treat forty patients with perioral and ocular wrinkles attributed to chronic photodamage [26]. At the 6-month follow-up, the average Fitzpatrick wrinkle score improved from 5.48 at baseline to 3.47, and most patients reported satisfaction with the improvement in wrinkles [26]. Therefore, the 755-nm alexandrite picosecond laser with DLA seems a good choice for treating wrinkles.

### Dyspigmentation disorders

Melasma is a pigmentation disorder related to photoaging. Chen et al. used a 755-nm alexandrite picosecond laser with DLA to treat 20 Asian patients with Fitzpatrick skin type IV with melasma. After treatment, the MASI scores showed a significant reduction and an improvement in the skin condition [27]. Wong et al. used fractional 1064-nm picosecond laser to treat melasma, and 70% patients showed moderate to marked improvement [28]. These results suggested the fractional picosecond laser with a DLA could be an effective treatment for melasma.

Post-inflammatory hyperpigmentation (PIH) is a common complication of inflammatory skin diseases [4]. In a study by Ren, they treated PIH patients with a 755-nm picosecond laser with DLA, and the pigmented lesions showed 50–75% improvement at the 3-year follow-up [4]. The results suggested the 755-nm picosecond laser with DLA could be a long-term effective treatment for PIH in Asian skin [4].

#### Enlarged facial pores

One study from Thailand used the 1064-nm picosecond laser with MLA to treat patients with enlarged pores. The results showed that after a single fractional picosecond 1064-nm laser treatment, the number of enlarged pores was 15.13% lower, while the diameter of the facial pores was unchanged [29]. In another study in Asians with enlarged pores treated with three monthly sessions of 1064-nm picosecond laser with MLA, there was a 30% decrease in average pore size at the 6-month follow-up [30]. The efficacy of fractional picosecond laser in the treatment of enlarged pores seems to need further study.

## Treatment for atrophic skin lesions

### Atrophic acne scars

Hypertrophic, atrophic, and keloid scars are the three different types of acne scars [15]. There are now several approaches to treat atrophic acne scars. Brauer et al. reported the efficacy of the 755-nm picosecond laser with DLA to treat atrophic acne scars [15]. They treated patients with a 755-nm picosecond laser with DLA (fluence of 0.71 J/cm^2^, 6 mm spot size, six sessions at 4- to 8-week intervals), and reported a mean scar improvement of 24.3% after the final treatment [15]. In a study by Dierickx et al., they treated 3 patients using the 755-nm picosecond laser with DLA, and they randomly assigned half of the face to be treated with the standard pulse coverage, while the other half of the face received 1.7 times more pulses [25]. The standard pulse side showed good to excellent improvement, while the high pulse side had very good to excellent improvement [25]. Huang reported that the 755-nm picosecond laser with DLA over several sessions was successful in treating atrophic acne scars in Asian patients [31]. A study by Zhang et al. [32] reported similar effects. Zhang treated 20 Chinese patients with a picosecond alexandrite laser with DLA over 3 treatment sessions, and suggested it effectively improved scar appearance and texture, without any serious adverse effects.

In Asian patients, a 1064-nm picosecond laser with MLA was used for treating atrophic acne scars. The skin surface roughness and the scars showed significant improvement, with the erythematous and hyperpigmented appearance improving at the same time [33]. Similarly, in patients from Thailand, the 1064-nm picosecond laser with MLA has also shown good results. All the patients’ acne scars were reduced significantly after one treatment session [29]. These clinical studies suggest that the fractional picosecond laser has a good effect on acne scars.

### Striae distensae

Striae distensae (or stretch marks) is another type of atrophic dermal scar, and is often caused by rapid changes in body weight, long-term exposure to steroids, or other endocrine conditions [34]. In a prospective study by Kaewkes, they treated twenty female patients with abdominal striae alba using a 1064-nm picosecond laser with a MLA handpiece using a fluence of 0.6 J/cm^2^, 8 mm spot size, over four sessions [34]. The results showed significant improvement in skin texture and striae, and only two patients developed PIH [34]. Fractional 1064-nm picosecond laser could be effective in treating striae distensae in dark skin types.

## Comparison of picosecond laser with other laser treatments

Tanghetti et al. compared the clinical and histological characteristics of skin lesions produced by a picosecond 532-nm and 1064-nm Nd:YAG laser with a holographic optic, or by a 755-nm picosecond laser with a DLA [35] (Table 2). Their study revealed that both devices caused skin vacuoles. The fractional 532-nm and 1064-nm picosecond laser caused cutaneous hemorrhages by damaging and heating the superficial blood vessels, while the 755-nm laser was absored by melanin and showed superior safety by avoiding any damage to the vasculature [35].

**Table 2:** Comparison of treatment

Disease	Laser	Therapy parameters	Fitzpatrick scale	Number of cases	Clinical outcome	Adverse events	Reference
Melasma	755‐nm picosecond laser with DLA and with a fullbeam	Coupled with a DLA on one side of the face and without DLA (flat optics) on the other side: 8‐mm spot, fluence of 0.40 J/cm^2^, 2.5 Hz for five treatments at 1‐month intervals	IV–V	18 (14 patients completed)	All patients had significant improvement in pigment clearance without significant differences in different treatment sides	Mild PIH and erythema	[36]
Acne scar	NAFL and P-DOE	P-DOE side: a 450-ps pulse duration, 2-dimensional 10 × 10 mm^2^ array of pulses, fluence of 130–430 mJ/cm^2^, 5–10 Hz, 10-mm spot size the NAFL side: 25–35 J/cm^2^ at level 4–6 over four to eight passes	III–IV III (*n* = 12); IV (*n* = 13)	25	P-DOE provides better clinical results, fewer side effects, and more improvements at the follow-up visit	Erythema, oedema, dryness; mild hyperpigmentation only on the NAFL side	[37]
Acne scar	The fractional Nd:YAG 1064-nm picosecond laser with FLA and fractional 1550-nm erbium fiber	The fractional Nd:YAG 1064-nm picosecond laser with FLA: spot size of 8 mm, fluence of 0.3–0.4 J, and frequency of 10 Hz for one pass; the fractional 1550-nm erbium fiber laser: 100–400 spots/cm^2^, energy of 25–30 J/cm^2^ for two passes total four times at 4-week intervals	III–IV III (*n* = 27); IV (*n* = 3)	30 (27 patients completed)	Median scores of both devices were significantly improved from baseline, and no significant difference between the two devices	Erythema, hyperpigmentation, pinpoint bleeding, pain	[38]
Acne scar	FxCO2 and FxPico	FxPico:8 mm spot size, a fluence of 0.8 J/cm^2^, 5 Hz repetitive rate; FxCO_2_: 3 ms pulse duration, a power of 10 W, depth of penetration about 350 to 400 mm	III–V	25	Both devices caused significant reduction in the scar volume without significant difference between them	Scarring, persistent erythema; PIH was only present in FxCO_2_ sides	[39]

A prospective split face study compared the 755-nm picosecond laser with DLA and the same laser used in a fullbeam mode for treating melasma. They found that all patients showed significant pigment clearance without any significant difference between the treatment sides. Compared to the fractional laser, the full-beam 755-nm picosecond laser showed a lower incidence of PIH, less downtime, and less pain during treatment [36].

A controlled study compared the effectiveness and safety of a non-ablative fractional laser (NAFL) or a 1064-nm Nd:YAG picosecond laser with a diffractive optical element (P-DOE) for treating acne scars [37]. In the treatment of acne scars in Asian patients, the P-DOE provided better clinical results, fewer side effects, and more improvement at the follow-up visit [37].

Similarly, another study compared the fractional Nd:YAG 1064-nm picosecond laser with a fractional lens array (FLA) with the fractional 1550-nm erbium fiber laser for treating acne scars [38]. The clinical scores with both devices showed significant improvement from baseline; however, there was no significant difference between the two devices. More pinpoint bleeding as a side effect was observed with the picosecond laser, whereas more pain was reported with the erbium laser [38].

Fractional carbon dioxide laser (FxCO_2_) was compared with fractional picosecond 1064-nm laser in a study to treat acne scars [39]. The results showed that FxCO_2_ was as effective as fractional picosecond 1064-nm laser, but the latter was accompanied by a lower incidence of PIH [39].

## Combinations of picosecond laser with other treatments

### Combination treatment for facial rejuvenation

Theoretically, soft tissue inflammation following laser treatment could affect the diffusion of botulinum neurotoxim to undesirable locations. Therefore, Wang et al. designed a study to test the combination of 755-nm picosecond laser with DLA plus botulinum toxin applied for facial rejuvenation [40]. There were no reported side effects from neurotoxin spreading, and the combined therapy could improve the efficacy with possible synergistic effects [40].

The 755-nm picosecond laser with DLA could treat wrinkles and fine lines, while the 1060-nm laser has been used for non-invasive lipolysis, and could improve the appearance of lax tissue [41]. Wang et al. combined the 755-nm picosecond laser with DLA with the 1,060-nm laser for submental lipolysis to treat patients with facial aging. Their results demonstrated that the combination of 1,060-nm laser submental lipolysis plus 755-nm picosecond laser with DLA could improve the overall clinical aesthetic effects [41].

Soft tissue fillers and laser treatments are frequently employed for facial rejuvenation [42]. Because laser treatment may change the filler properties, patient safety and filler efficacy could be a concern. Therefore, Wang et al. designed a retrospective study and found that there were no recorded adverse effects among 406 single-session treatments in patients receiving 755-nm picosecond laser with DLA combined with fillers [42].

### Combination treatments for benign pigmented lesions

In a split face study by Chalermchai, they treated melasma patients with fractional picosecond 1064-nm laser plus 4% hydroquinone on the intervention side, and 4% hydroquinone cream alone on the control side[43]. The intervention side showed greater reductions in melasma area severity index scores compared to 4% hydroquinone cream alone, and it was suggested that fractional picosecond 1064-nm laser could be an effective treatment for melasma.

Various methods have been tested to remove unwanted tattoos, and there was a study to compare tattoo removal results with fractionated 1064-nm picosecond laser plus unfractional 1064-nm picosecond laser, with unfractional 1064-nm picosecond laser alone [44]. The combination side showed greater clearance scores and fewer adverse events than the unfractional 1064-nm picosecond laser alone; as a result, they suggested that combination therapy may be a good method for tattoo removal [44].

### Combination treatments for scars

Kim combined the 1064-nm picosecond laser with MLA and hyaluronic acid fillers (HAF) to treat acne scars and achieved good results. Their histological findings suggested that the laser did not disrupt the pre‐injected HAF, and also induced significant neocollagenesis [45].

In a study by Feng et al., an intense pulsed light (IPL) device was used on its own to treat one-half of the face, while the other half was treated by fractional 1064-nm Nd:YAG picosecond laser (FxPico) combined with IPL. The combination showed greater pore count reduction and scar improvement, and they suggested that FxPico combined with IPL could be a better treatment option for atrophic acne scars [46].

Rho et al. reported a patient with a full-thickness laceration scar who was treated with 1064-nm Nd:YAG laser with MLA combined with polynucleotide gel injection, and reported a significant clinical improvement [47].

## Conclusion

**Fig. 2:**
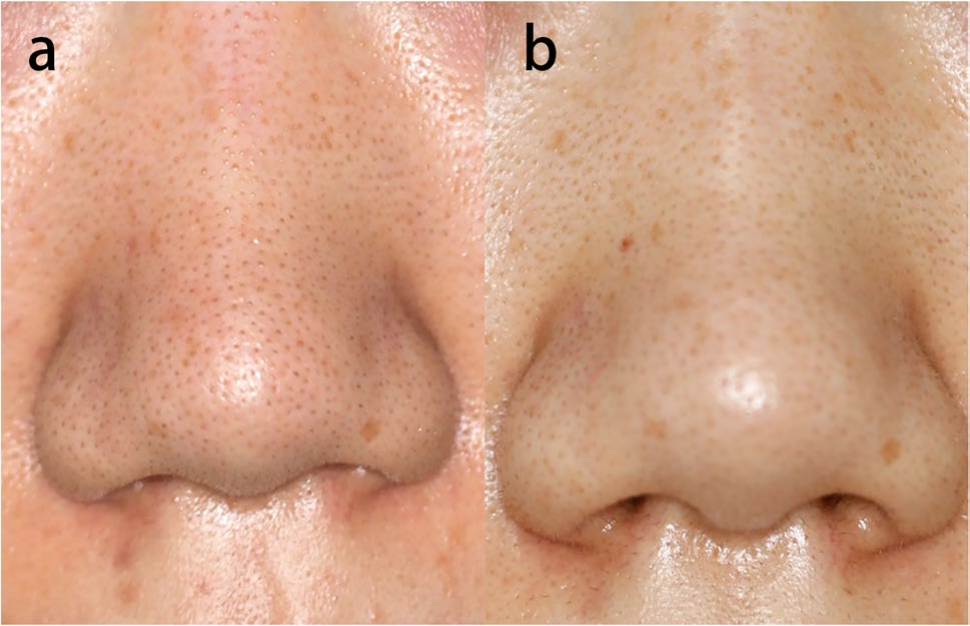
**A** Photo of a patient with enlarged facial pores; **b** 2 months after one fractional picosecond laser treatment (wavelength 755 nm, energy 0.4 J/cm.^2^, frequency 5 Hz, spot size 8 mm)

Clinical studies have reported that the high-intensity micro-injury zones caused by fractional picosecond laser can improve atrophic scars, enlarged facial pores, dyspigmentation, and wrinkles (Fig. 2). Combination therapy with fractional picosecond laser does not affect the efficay of other treatments, and may show additive or synergistic benefits (Table 3). More clinical research on different disorders treated with fractional picosecond laser is needed to improve our understanding of the overall benefits of fractional picosecond laser.

**Table 3:** Combination therapy of fractional picosecond lasers

Disease	Laser	Therapy parameters	Fitzpatrick scale	Number of cases	Clinical outcome	Adverse events	Reference
Facial rejuvenation	755-nm picosecond laser with DLA and botulinum toxin	Botulinum toxin: mean total units per treatment was 39.5 units	I–V I (*n* = 75); II (*n* = 76); III (*n* = 45); IV (*n* = 11); V (*n* = 1)	208	Combined therapy improved efficacy and introduce synergistic effects	No adverse events recorded	[40]
	755 nm picosecond laser with DLA and 1060 nm laser lipolysis	755-nm picosecond laser with DLA: fluence of 0.71 J/cm^2^, 6 mm spot size for 3 Laser treatments and 2 lipolysis treatments at 2- to 8-week intervals	II–VI	11	Neck laxity improved in all subjects from baseline	Transient pain, nodule formation, dyspigmentation, tenderness, edema, roughness, and blister formation	[41]
	Facial fillers and 755-nm picosecond laser with DLA	Soft tissue fillers; picosecond 755-nm alexandrite laser with DLA: the mean was 3,730.2 pulses	I–IV I (*n* = 71), II (*n* = 64), III (*n* = 38),IV (*n* = 10)	183	Single-session facial fillers and 755-nm picosecond laser with DLA was safe	No adverse events recorded	[42]
Melasma	Fractional picosecond 1064-nm laser and 4% hydroquinone	Fractional picosecond 1064-nm laser: fluence of 1.3–1.5 mJ per microbeam, pulse duration 450 ps, 4 Hz; daily application of 4% hydroquinone	III–IV III (*n* = 5), IV (*n* = 25)	30	The intervention side considerably reduced the melasma area severity index scores than 4% hydroquinone cream alone	Mild erythema,skin desquamation and burning sensation	[43]
Tattoo	Fractionated 1064-nm picosecond lasers and unfractional 1064-nm picosecond lase	The 1064-nm picosecond laser: fluence of 1.5–7.24 J/cm^2^; spot size: 3–4.5 mm, 2- 5 Hz; fractionated 1064-nm picosecond lasers: fluence of 0.8 J/cm^2^; spot size: 8 mm, 2–5 Hz	III–V III–IV (*n* = 8) V (*n* = 3)	11	The combination side showed greater clearance scores and fewer adverse events than the side of unfractional 1064-nm picosecond laser alone	Temporary crusting, purpura, edema, erythema, burning, sensation, and petechiae	[44]
Acne scars	1064-nm MLA‐type picosecond lasers and HAF	Each scar was filled with 0.01–0.1 ml of HAF and MLA handpiece: 6 mm spot, fluence of 1.4 J/cm^2^, 5 Hz frequency; 450 ps pulse duration for two treatments at 4‐week intervals	III–IV	36	Acne scars improved significantly	Temporary pain, instant erythema, and flushing	[45]
	FxPico and IPL	FxPico: 6 mm spot size, energy of 1.5–2.5 mJ/microbeam, 3–4 passes IPL: 560- or 590-nm filter, pulse width of 3.5–4.0 ms, fluence of 15–19 J/cm^2^, 1 pass One half of the face treated by FxPico + IPL, and the other by IPL alone for five sessions of treatment	III–IV III (*n* = 13); IV (*n* = 2)	17 (15 patients completed)	More pore count reduction and scar improvement were observed on the FxPico + IPL side	Mild‐to‐moderate pain, erythema, edema, petechiae, crusting, pruritus, and acneiform eruptions,	[46]
Full-thickness laceration scar	1064-nm Nd:YAG laser with MLA and polynucleotide gel	1064-nm Nd:YAG laser with MLA at fluence of 0.7 J/cm^2^, and then 20 mg/mL polynucleotide gel was injected	Not mentioned	1	Significant clinical improvement	pruritus	[47]

## Data Availability

The data used to support the findings of this study are available from the corresponding author upon request.

## Abbreviations

*Nd: YAG*: Neodymium: yttrium‐aluminum‐garnet
*CO*_*2*_: Carbon dioxide
*Er: YAG*: Erbium: yttrium–aluminum-garnet
*DLA*: Diffractive lens arrays
*MLA*: Micro-lens arrays
*LIOB*: Laser-induced optical breakdown
*LIC*: Laser-induced cavitation
*PIH*: Post-inflammatory hyperpigmentation
*NAFL*: Non-ablative fractional laser
*P-DOE*: Picosecond laser with a diffractive optical element
*FLA*: Fractional lens array
*FxCO*_*2*_: Fractional carbon dioxide laser
*HAF*: Hyaluronic acid fillers
*IPL*: Intense pulsed light
*FxPico*: Fractional 1064-nm Nd: YAG picosecond laser
